# Development and Validation of the ClimHealth‐K: A Scale for Assessing Perceived Knowledge and Awareness of Climate Change‐Related Health Effects

**DOI:** 10.1111/nhs.70350

**Published:** 2026-05-07

**Authors:** Taha Gökmen Ülger, Elif Gençer Şendur, Esra Tunçer, İsa Çelik, Murat Bektaş

**Affiliations:** ^1^ Department of Nutrition and Dietetics Bolu Abant Izzet Baysal University Bolu Türkiye; ^2^ Department of Nursing Bolu Abant Izzet Baysal University Bolu Türkiye; ^3^ Department of Nursing Recep Tayyip Erdoğan University Rize Türkiye; ^4^ Department of Nursing Dokuz Eylül University Izmir Türkiye

**Keywords:** awareness, climate change, perceived knowledge, psychometric validation, public health, scale development

## Abstract

Climate change poses a significant threat to human health and is linked to various direct and indirect outcomes, including infectious diseases, heat‐related illnesses, and other climate‐sensitive conditions. However, standardized tools assessing perceived knowledge and awareness of climate‐related health effects remain limited. This methodological study aimed to develop and validate the ClimHealth‐K scale for assessing university students' perceived climate‐health knowledge. The sample included 449 students from a public university in Türkiye. Items were generated through literature review and evaluated by seven experts to establish content validity using the Davis method. Psychometric analyses included exploratory and confirmatory factor analyses and reliability testing. Item‐level content validity index values ranged from 0.86 to 1.00, and the scale‐level index was 0.98. Exploratory factor analysis supported a unidimensional structure explaining 62.67% of total variance, and confirmatory factor analysis showed good model fit (*χ* ^2^/df = 2.27, CFI = 0.977, RMSEA = 0.075). Cronbach's alpha was 0.954, and test–retest reliability indicated moderate stability (ICC = 0.653, *p* < 0.001). The 11‐item ClimHealth‐K is a valid and reliable instrument for assessing perceived climate‐health knowledge in educational and public health research.

## Summary

- What this paper adds
  ○ Introduces the ClimHealth‐K, a newly developed and psychometrically validated scale for assessing perceived knowledge and awareness of the health effects of climate change among university students.
  ○ Demonstrates that the ClimHealth‐K is a valid and reliable single‐factor, 11‐item instrument with strong internal consistency and acceptable test–retest reliability.
  ○ Provides a practical tool for researchers, educators, and policymakers to identify gaps in perceived knowledge and awareness, design targeted interventions, and support evidence‐based climate adaptation strategies in public health.

## 1 Introduction

Climate change is recognized as one of the most pressing global challenges of the 21st century and poses substantial risks to human health by affecting the physical environment, ecosystems, socioeconomic structures, and the sustainability of healthcare systems (World Health Organization [WHO] 2023). The increasing severity and complexity of climate‐related health threats have resulted in profound public health challenges (Romanello et al. 2024). Climate change is closely associated with rising temperature‐related mortality as well as a higher incidence of food‐, water‐, and vector‐borne infectious diseases. Mental health disorders are also negatively affected by climate change, while additional threats such as floods, biodiversity loss, and declining agricultural productivity further exacerbate environmental and human vulnerabilities (Intergovernmental Panel on Climate Change [IPCC] 2023). According to WHO estimates, between 2030 and 2050, climate change will cause approximately 250 000 additional deaths each year due to heat stress, diarrhea, malaria, and undernutrition. Furthermore, the direct health costs attributable to climate change are projected to reach between $2 and $4 billion annually by 2030 (WHO 2023).

The health consequences of climate change occur through both direct and indirect pathways (Weber et al. 2023). Meteorological factors such as temperature, precipitation, humidity, wind, and extreme weather events significantly influence the dynamics of infectious disease transmission. Floods and other major water‐related events are linked to an increased risk of vector‐borne diseases. In addition, high temperatures and humidity, combined with reduced precipitation, are associated with food‐ and waterborne illnesses such as cholera, schistosomiasis, Salmonella infections, and *E. coli* gastroenteritis (Rocque et al. 2021). Environmental stressors including extreme heat and hurricanes increase cardiovascular morbidity and mortality (Kazi et al. 2024). Drought increases dust levels, negatively affecting both cardiovascular and respiratory health. Exposure to air pollutants and wildfire smoke is associated with asthma, chronic obstructive pulmonary disease, and reduced lung function (Rocque et al. 2021). Additionally, climate‐driven alterations in allergen exposure contribute to a higher prevalence and severity of allergic diseases, thereby placing additional strain on healthcare services (Montoro et al. 2024; Rida et al. 2024). Extreme weather events have also been linked to greater demand for healthcare services, including emergency visits, hospital admissions, and ambulance utilization, while power outages and infrastructure disruptions may further compromise healthcare delivery (Rocque et al. 2021).

Although several instruments have been developed to assess various aspects of the relationship between climate change and health, existing tools differ considerably in their conceptual focus, target populations, and intended outcomes. Most available scales primarily address multidimensional constructs such as climate change health literacy, professional perceptions, protective behaviors, or psychological responses to climate change (Clayton and Karazsia 2020; Innocenti et al. 2021; Kurt et al. 2024; Nayir et al. 2025; Sönmez et al. 2025). However, there is a lack of concise instruments specifically designed to measure individuals' self‐perceived knowledge and awareness of the health effects of climate change as a distinct cognitive construct, particularly among university students. Therefore, the Scale for Assessing Perceived Knowledge and Awareness of Climate Change‐Related Health Effects (ClimHealth‐K) was developed to address this methodological and conceptual gap by providing a brief, unidimensional, and psychometrically robust tool that focuses exclusively on perceived knowledge and awareness, which represent critical antecedents of attitude formation and health‐protective behaviors.

The broad and escalating health consequences of climate change highlight the urgent need for well‐informed professionals who can take on leadership roles in society, contribute to sustainable planning of living environments, influence nutrition and lifestyle practices, shape occupational and working conditions, and effectively communicate scientific evidence to the public. Enhancing the perceived knowledge and awareness of university students, who represent the future decision‐makers and health professionals, is essential to building societal resilience and enhancing public health preparedness (Yörük and Akpinar 2023).

The present study was conducted with the aim of developing a valid and reliable instrument to assess perceived knowledge and awareness regarding the health effects of climate change among university students. In line with this aim, the study sought to address the following research question: To what extent does the ClimHealth‐K demonstrate robust psychometric properties, including validity and reliability, when measuring perceived knowledge and awareness of the health‐related consequences of climate change?

## 2 Methods

**FIGURE 1:**
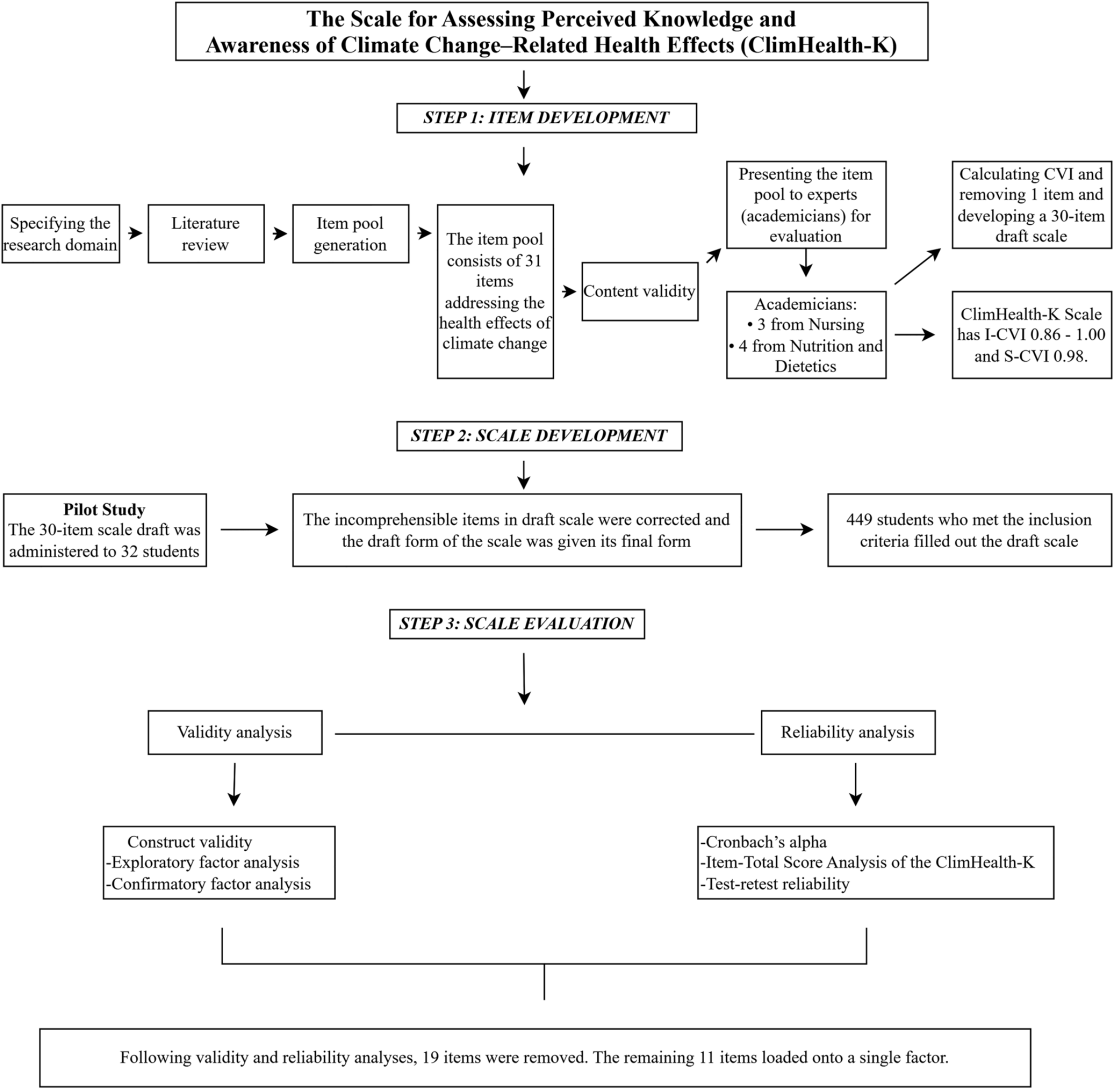
Study design flowchart.

This study was designed as a methodological study and was conducted in three distinct phases. These phases included: (1) determining the conceptual framework and generating the item pool of the scale, (2) conducting the pilot and main implementations of the scale, and (3) testing the psychometric properties and finalizing the scale (Figure 1).

### 2.1 Phases of the Study

#### 2.1.1 Phase 1: Determining the Conceptual Framework and Generating the Item Pool

A comprehensive literature review was conducted to establish the conceptual structure of the measurement tool. The databases PubMed, ScienceDirect, and Google Scholar were searched using the keywords “climate,” “climate change,” “climate crisis,” “health,” and “health impact,” without any date restrictions. According to the study, the majority of the research that has been done on the connection between climate change and health has been on how it affects temperature and air quality, infectious and chronic diseases, nutrition, migration, socioeconomic and psychological implications, and vulnerable groups. Accordingly, the initial item pool was developed to reflect all of these thematic areas.

To enhance transparency in the item development process, the retrieved literature was screened in a structured manner. Studies were included if they addressed the health impacts of climate change in human populations and were published in peer‐reviewed journals. Editorials, commentaries, and studies focusing exclusively on environmental, ecological, or economic outcomes without explicit reference to human health were excluded. The reviewed literature consistently emphasized key thematic domains such as temperature‐related health outcomes, air pollution and respiratory diseases, food and water safety, infectious diseases, mental health effects, vulnerable populations, and indirect socioeconomic and nutritional consequences of climate change. These recurring themes informed the conceptual framework of the scale and guided the generation of the initial item pool, ensuring comprehensive content coverage and conceptual relevance.

Based on the literature findings, three researchers collaboratively created a draft item pool. In line with the guidelines proposed by DeVellis and Thorpe (2021), items that differed by only a single word were retained in the initial pool to preserve content variation. The preliminary pool consisted of 32 items. This item set was then reviewed by five experts for relevance, coherence, and importance, resulting in a revised version containing 31 items.

At the final stage of this phase, the revised draft scale, now consisting of 31 items, was once again evaluated for content validity and pilot testing. Content validity was assessed using the Content Validity Index (CVI). Three nursing experts and four nutrition and dietetics experts made up the panel of seven academic experts who assessed each topic on a four‐point scale: 1 representing “not relevant,” 2 “somewhat relevant (major revision needed),” 3 “quite relevant (minor revision needed),” and 4 “highly relevant.” The Item‐Level Content Validity Index (I‐CVI) was calculated based on the proportion of experts who rated each item as either 3 or 4. The I‐CVI threshold of 0.80 was established as the lowest acceptable value (Davis 1992). With an I‐CVI of 0.71, below the cutoff, one item (*Climate change increases anxiety about the future*) was removed from the scale's final edition.

#### 2.1.2 Phase 2: Pilot Testing and Main Implementation of the Scale

According to the literature, scales that have achieved content validity should be tested on a group similar to the target sample, consisting of approximately 30 to 100 individuals (Kishore et al. 2021). The 30‐item scale, which had been modified based on the CVI, was given to 32 university students as part of a pilot test to assess item clarity, comprehensibility, and ease of completion. Participants were asked whether any items were difficult to understand or respond to. This process aimed to identify potential issues related to wording and interpretation. Two items were changed based on the input received, using more commonly known phrases in place of less familiar expressions. To improve clarity and comprehensibility, the term “stroke” was replaced with “paralysis,” and the expression “exposure” was reworded as “being exposed.”

#### 2.1.3 Phase 3: Testing Psychometric Properties and Finalizing the Scale

In this phase, the validity and reliability of the scale were evaluated.

### 2.2 Sample and Data Collection

The population of the study consisted of undergraduate students enrolled at Bolu Abant İzzet Baysal University. The sample included students from the university's undergraduate programs who met the inclusion criteria, which were voluntary participation, being 18 years of age or older, current undergraduate enrollment, and having no visual or auditory impairments. Students who did not meet these criteria or who did not agree to participate voluntarily were excluded from the study. Data were collected between November 2024 and January 2025 using an online questionnaire administered via Google Forms. The survey link was disseminated through general institutional communication channels as an open invitation to undergraduate students, and participation was entirely voluntary. Because the invitation was not distributed to a predefined sampling frame, the exact number of students who received or viewed the survey link could not be determined. Prior to analysis, incomplete or invalid questionnaires were excluded, resulting in a final analytic sample of 449 undergraduate students.

In scale development research, it is recommended that exploratory factor analysis (EFA) and confirmatory factor analysis (CFA) be conducted with at least 200 and 400 participants, respectively (Boateng et al. 2018; Hair et al. 2018). These recommendations were met in the present study. In addition, a subset of 41 participants from the final sample completed the questionnaire a second time for the purpose of test–retest reliability assessment.

### 2.3 Data Collection Instruments

In addition to the 30‐item ClimHealth‐K scale, participants were asked to complete a 7‐item personal information form. The Personal Information Form consisted of seven items that gathered data on socio‐demographic characteristics such as age and gender, educational background including faculty and year of study, as well as participants' familiarity with the concept of climate change and their related opinions and attitudes.

ClimHealth‐K is a five‐point Likert‐type scale developed by the authors. The response options were as follows: 1 = Strongly disagree, 2 = Disagree, 3 = Neither agree nor disagree, 4 = Agree, and 5 = Strongly agree. A higher total score on the scale indicated a higher level of perceived knowledge and awareness regarding the health impacts of climate change.

### 2.4 Data Analysis

For validity analyses, the Davis technique, EFA, and CFA were employed. To divide the total sample (*n* = 449) into two independent subsamples for exploratory factor analysis (EFA; *n* = 225) and confirmatory factor analysis (CFA; *n* = 224), an order‐based allocation procedure was applied. Specifically, participants were assigned to subsamples according to their order in the dataset: cases with odd sequence numbers were allocated to the EFA group, whereas cases with even sequence numbers were allocated to the CFA group. This procedure ensured transparency and reproducibility of the sample split while yielding two independent and comparable subsamples. In the EFA phase, Principal Axis Factoring was utilized as the extraction method, and Direct Oblimin was applied as the rotation method.

The model fit in the CFA was assessed using several goodness‐of‐fit indices. These included the chi‐square to degrees of freedom ratio (*χ* ^2^/df), Goodness of Fit Index (GFI), Adjusted Goodness of Fit Index (AGFI), Comparative Fit Index (CFI), Incremental Fit Index (IFI), Tucker‐Lewis Index (TLI), Normed Fit Index (NFI), and Root Mean Square Error of Approximation (RMSEA).

Reliability analyses were carried out using Cronbach's alpha coefficient, item‐total correlations, and the test–retest method. These analyses were used to evaluate the internal consistency and temporal stability of the scale.

Ceiling effects were examined to evaluate the scale's ability to discriminate among higher levels of knowledge, with a threshold of 15% used to indicate the presence of a ceiling effect.

## 3 Results

A total of 449 participants, including 112 males and 337 females, took part in the study. The mean age of the participants was 20.63 ± 2.40 years, and the age range was between 18 and 42 years. Nearly half of the participants were students from the Faculty of Health Sciences (49.7%), followed by students from the Faculty of Dentistry (20.3%), Faculty of Education (10.5%), Faculty of Sports Sciences (10%), and Faculty of Arts and Sciences (3.8%). Additionally, 60.8% of the participants were first‐ or second‐year undergraduate students (Table 1).

**TABLE 1:** Characteristics of the participants (*n* = 449).

Characteristics			
Age (Mean ± SD)		20.63	2.4

		Frequency	Percentage
Gender	Female	337	75.1
	Male	112	24.9
Faculty of enrollment	Health Sciences	223	49.7
	Dentistry	91	20.3
	Education	47	10.5
	Sport Sciences	45	10
	Arts and Sciences	17	3.8
	Other	26	5.7
Year of study	Freshman	145	32.3
	Sophomore	128	28.5
	Junior	91	20.3
	Senior	85	18.9
Awareness of the concept of climate change	Aware (of the concept of climate change)	425	94.7
	Not aware	24	5.3
Belief that climate change affects human health	Believe (that climate change affects human health)	435	96.8
	Do not believe	14	3.1
Source(s) of information about climate change	Media	376	83.7
	Courses/Seminars/Scientific Publications	73	16.3

A majority of the participants (94.7%) reported having previously heard of the concept of climate change, and 96.9% believed that climate change could have an impact on human health. The most commonly cited source of information was the media (83.7%), while 16.3% stated that they had attended a course or congress related to climate change (Table 1).

### 3.1 Content Validity

According to the results of the content validity assessment, the I‐CVI values ranged from 0.86 to 1.00, while the Scale‐Level Content Validity Index (S‐CVI) was calculated as 0.98.

### 3.2 Exploratory Factor Analysis

In the present study, the Kaiser‐Meyer‐Olkin (KMO) value was found to be 0.947, and Bartlett's test result was *χ* ^2^ = 1903.491, with a significance level of *p* < 0.001, indicating that the data were suitable for factor analysis. The factor structure was explored using Principal Axis Factoring (PAF) as the extraction method and Direct Oblimin as the rotation method. The analysis supported a unidimensional structure of the scale. EFA revealed a single factor that accounted for 62.67% of the total variance. Nineteen items with factor loadings below the criterion value of 0.32 were removed from the scale. The factor loadings of the items ranged between 0.716 and 0.867, with all loadings above the acceptable threshold of 0.32 (Table 2).

**TABLE 2:** Exploratory factor analysis of scale (*n* = 225).

Items		Factor
Item 1	Climate change poses greater risks to individuals with chronic diseases (e.g., obesity, heart failure, diabetes).	0.729
Item 2	Temperature fluctuations associated with climate change increase emergency department visits.	0.797
Item 3	Climate change increases healthcare expenditures.	0.746
Item 4	Climate change increases the risk of excessive fluid loss.	0.784
Item 5	Climate change increases the risk of cardiovascular diseases.	0.830
Item 6	Climate change poses greater risks to individuals with allergies.	0.867
Item 7	Climate change increases the incidence of infectious diseases (e.g., cholera).	0.716
Item 8	Climate change increases the distribution and prevalence of certain disease vectors (e.g., mosquitoes, ticks).	0.843
Item 9	Climate change increases the risk of cancer.	0.763
Item 10	Climate change increases the prevalence of respiratory diseases (e.g., asthma, bronchitis).	0.829
Item 11	Extreme weather events associated with climate change (e.g., hurricanes, floods) increase the incidence of physical injuries.	0.789
	Explained total variance (%)	62.67
	KMO coefficient	0.947
	Bartlett test	< 0.001
	Extraction Method: Principal Axis Factoring	
	Rotation: Direct Oblimin	

### 3.3 Confirmatory Factor Analysis

**FIGURE 2:**
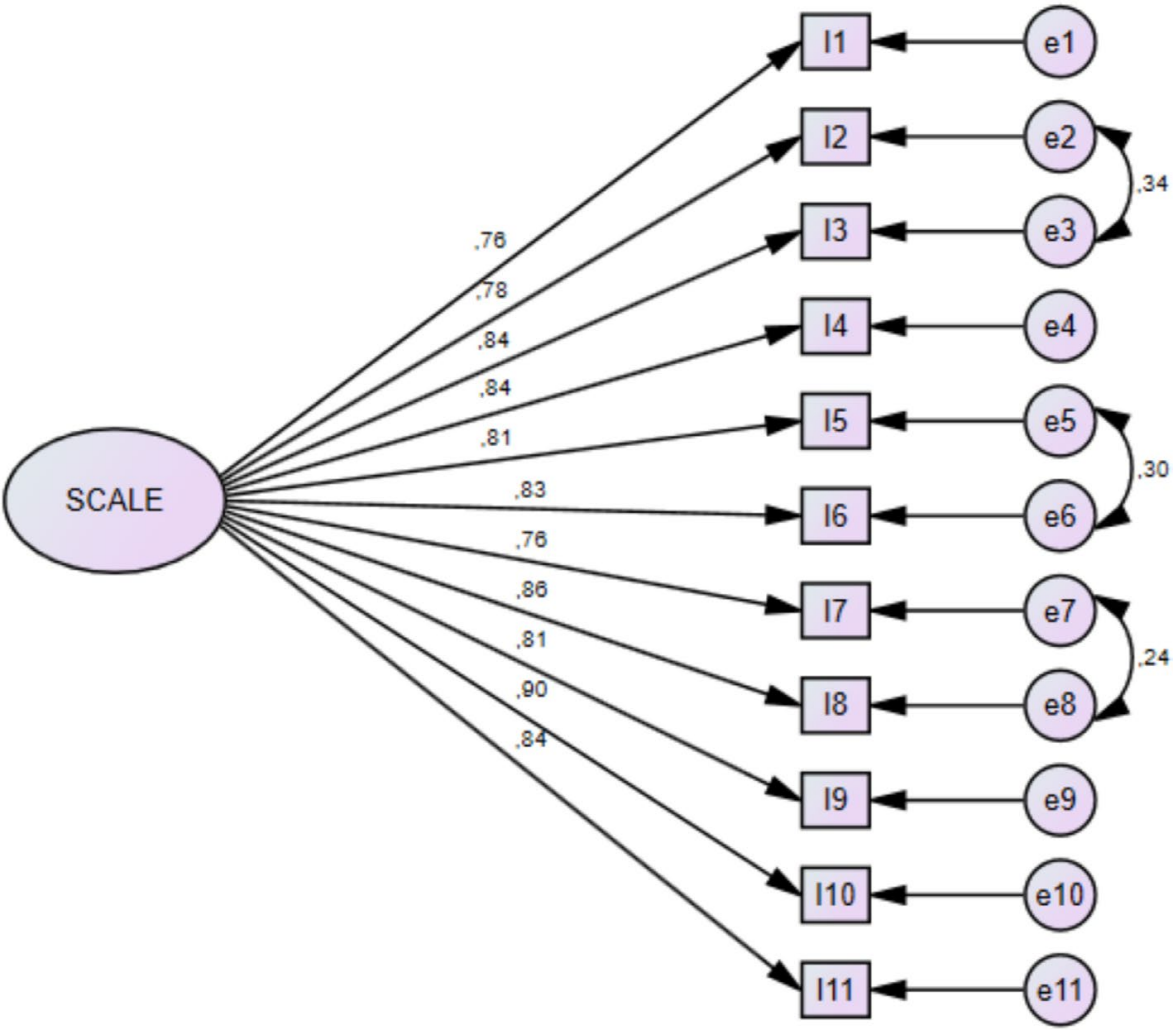
Confirmatory factor analysis.

The results of the confirmatory factor analysis indicated that the single‐factor model demonstrated an acceptable level of fit (Figure 2). The model fit indices were as follows: *χ* ^2^/df = 2.270, GFI = 0.930, AGFI = 0.888, CFI = 0.977, IFI = 0.977, TLI = 0.968, NFI = 0.959, and RMSEA = 0.075. Detailed results are presented in Table 3.

**TABLE 3:** Model fit indices from confirmatory factor analysis (*n* = 224).

Model/Data‐model fit indices	AGFI	NFI	*χ* ^2^/df	RMSEA	CFI	IFI	TLI	GFI
One‐factor model	0.888	0.959	2.270	0.075	0.977	0.977	0.968	0.930

Abbreviations: AGFI, adjusted goodness of fit index; CFI, comparative fit index; df, degrees of freedom; GFI, goodness of fit; IFI, incremental fit index; NFI, normed fit index; RMSEA, root mean standard error approximation; TLI, Tucker–Lewis index; *χ* ^2^, Chi‐square.

The scale's sensitivity in distinguishing between different levels of knowledge was further evaluated through ceiling effect analysis. Regarding the ceiling effect, 38 participants (8.46%) achieved the maximum possible score of 55.

### 3.4 Reliability Analyses

#### 3.4.1 Internal Consistency and Descriptive Results

Item‐level descriptive statistics for the scale, Cronbach's Alpha, If Item Deleted, Corrected Item‐Total Correlation, and Scale Variance if Item Deleted are presented in Table 4. It was determined that the correlations of the scale items with the scale total score ranged between 0.723 and 0.840. The Cronbach's alpha value was 0.954 for the entire scale. When removed from the scale, it was determined that no item significantly increased Cronbach's alpha. The mean scores for the individual items ranged from 3.84 ± 0.992 to 4.16 ± 0.968. Analysis of the total scale score (*n* = 449) revealed a mean of 44.31 (SD = 8.93), a skewness value of −1.729, and a kurtosis value of 3.866. The observed total scores ranged from 11 to 55.

**TABLE 4:** Reliability analysis results of the scale (*n* = 449).

Item	Mean ± SD	Cronbach's Alpha if item deleted	Corrected item‐total correlation (*r*)^⁎^	Scale variance if item deleted
Item 1	3.93 ± 0.938	0.952	0.723	67.747
Item 2	4.03 ± 0.971	0.950	0.773	66.602
Item 3	4.05 ± 0.993	0.950	0.784	66.138
Item 4	4.07 ± 0.980	0.950	0.789	66.253
Item 5	3.95 ± 0.983	0.949	0.806	65.959
Item 6	4.12 ± 0.996	0.948	0.832	65.399
Item 7	3.84 ± 0.992	0.952	0.732	66.928
Item 8	4.16 ± 0.968	0.948	0.834	65.766
Item 9	4.00 ± 1.008	0.951	0.764	66.244
Item 10	4.11 ± 0.982	0.948	0.840	65.470
Item 11	4.06 ± 0.976	0.950	0.793	66.246

*Note:* Cronbach's alpha 0.954.

#### 3.4.2 Test‐Retest

To assess the temporal stability of the scale, a test–retest procedure was conducted. Test–retest reliability was evaluated in a convenience subsample of 41 participants who were voluntarily recruited from the original study sample. The scale was re‐administered to these participants after a 2‐week interval. Test–retest reliability was assessed using a two‐way mixed‐effects model Intraclass Correlation Coefficient (ICC) based on single measures and absolute agreement. The ICC for single measures was 0.653, with a 95% confidence interval of [0.437, 0.798], indicating moderate temporal stability. The result was statistically significant (*p* < 0.001). In addition, a paired‐samples *t*‐test was conducted to compare the mean scores obtained at Time 1 (*M* = 48.39, SD = 4.51) and Time 2 (*M* = 49.41, SD = 5.03). The difference between the two measurements was not statistically significant, *t*(40) = −1.67, *p* = 0.103, further supporting the stability of the scale over time.

## 4 Discussion

The present study developed and psychometrically evaluated the Scale for Assessing Perceived Knowledge and Awareness of Climate Change, ClimHealth‐K, as a self‐reported instrument designed to measure perceived knowledge and awareness of the health effects of climate change among university students. A key contribution of this study is the development of a concise, unidimensional, and psychometrically robust measurement tool that focuses on perceived climate and health knowledge, a cognitive construct that has received comparatively limited attention in existing measurement approaches.

Climate change is increasingly recognized as a major global public health challenge with wide‐ranging direct and indirect effects on human health. Understanding how individuals perceive their knowledge and awareness of these health‐related consequences is essential, as such perceptions influence risk appraisal, motivation to seek information, and engagement in health‐protective behaviors. In this context, standardized and reliable quantitative instruments are required to assess perceived climate and health knowledge in educational, public health, and policy‐oriented research.

The findings of the present study indicate that the ClimHealth‐K demonstrates sound psychometric properties. The scale showed strong evidence of content validity, a clear single‐factor structure supported by both exploratory and confirmatory factor analyses, high internal consistency, and satisfactory temporal stability. Together, these findings suggest that the ClimHealth‐K provides a valid and reliable means of assessing perceived climate health knowledge and awareness.

### 4.1 Content Validity

In this study, the relevance and appropriateness of the scale items were evaluated by seven experts. The literature suggests that obtaining opinions from 3 to 20 experts is recommended for content validation (DeVellis and Thorpe 2021; Johnson and Christensen 2024). Using the Davis technique, the CVI was calculated for each of the 30 items. The (I‐CVI values ranged from 0.86 to 1.00, while the S‐CVI was determined to be 0.98. A value of 0.80 or higher is generally considered the threshold for acceptable content validity (Davis 1992). Accordingly, the S‐CVI score of 0.98 provides strong evidence supporting the content validity of the scale. These findings indicate that each item included in the scale demonstrated adequate content validity.

Nevertheless, several limitations related to the content validity process should be acknowledged. Although the number of experts involved in the evaluation met the range recommended in the literature, the expert panel was limited to the fields of nursing and nutrition and dietetics. As a result, perspectives from complementary disciplines such as public health, environmental health, psychology, and measurement and evaluation may not have been sufficiently represented during the content validation stage. This limitation may have constrained the comprehensive coverage of certain dimensions of climate change–health knowledge. Future studies are therefore encouraged to include a broader and more interdisciplinary group of experts to further strengthen the content scope and validity of the scale.

### 4.2 Construct Validity

#### 4.2.1 Exploratory Factor Analysis

The suitability of the data for factor analysis and the adequacy of the sample size were assessed using Bartlett's Test of Sphericity and the KMO measure. In the present study, the KMO value was 0.947 and Bartlett's Test was statistically significant, indicating that the data were suitable for factor analysis.

Exploratory factor analysis was conducted using an iterative item evaluation and reduction process consistent with recommendations in the scale development literature. Items with low factor loadings, substantial cross‐loadings, or limited conceptual relevance were identified and removed in a stepwise manner. The analysis was repeated after each removal until a clear and stable factor structure was achieved.

The exploratory factor analysis revealed a unidimensional structure for the ClimHealth‐K, with a single factor explaining 62.67% of the total variance. This proportion of explained variance exceeds commonly accepted minimum thresholds (DeVellis and Thorpe 2021; McDonald 2013) and indicates strong structural validity. Beyond its statistical adequacy, the emergence of a clear single factor solution has important conceptual implications. The unidimensional structure may indicate that perceived knowledge and awareness regarding the health effects of climate change operate as a relatively coherent cognitive domain among university students, suggesting that climate‐related health information tends to be integrated rather than processed as distinct subdomains. From a practical perspective, the unidimensional structure enhances the interpretability and usability of the scale in educational and public health contexts.

#### 4.2.2 Confirmatory Factor Analysis

In the present study, the chi square to degrees of freedom (*χ* ^2^/df) ratio was below three, indicating that the single factor model demonstrated an acceptable level of fit. In addition, CFI, IFI, TLI, and NFI values exceeded 0.95, indicating good model fit. The AGFI value of 0.888 falls within the acceptable range, further supporting the adequacy of the model (Schermelleh‐Engel et al. 2003). Similarly, the RMSEA value obtained in this study lies within the recommended range and indicates sufficient fit of the model to the observed data.

Importantly, the collective pattern of fit indices indicates not only statistical adequacy but also conceptual consistency between the proposed single factor model and the underlying construct. The acceptable to good fit observed across multiple indices suggests that the unidimensional structure adequately captures the latent construct of perceived knowledge and awareness regarding the health effects of climate change. Overall, the CFA results support the compatibility of the proposed structure with the data and provide evidence for the construct validity of the ClimHealth‐K.

The instrument's sensitivity was supported by the absence of a significant ceiling effect. According to Terwee et al. (2007), a ceiling effect is considered present when more than 15% of respondents attain the maximum possible score. In the present study (*n* = 449), only 8.46% of participants (*n* = 38) achieved the maximum score of 55. This finding indicates that the scale demonstrates adequate discriminative capacity and effectively differentiates among higher levels of perceived knowledge without compromising measurement reliability (Terwee et al. 2007).

### 4.3 Reliability

To evaluate the reliability of the ClimHealth‐K scale, which is structured as a five‐point Likert‐type scale, Cronbach's alpha reliability coefficient was calculated. A minimum acceptable Cronbach's alpha coefficient of 0.70 has been suggested in the literature (DeVellis and Thorpe 2021). The overall Cronbach's alpha coefficient for the scale was 0.954, which exceeds the recommended threshold and indicates high internal consistency in measuring perceived knowledge and awareness regarding the health effects of climate change.

Although alpha values above 0.90 may sometimes indicate potential item redundancy (DeVellis and Thorpe 2021), in the present study high alpha values are considered appropriate given the narrowly defined and unidimensional nature of the construct. This interpretation is supported by the high factor loadings and satisfactory item total correlations observed for all retained items, suggesting that the items capture related but conceptually nonredundant aspects of perceived climate health knowledge. The convergence of internal consistency estimates, factor loadings, and item–total correlations indicated that all retained items contributed meaningfully to the construct; therefore, no further item reduction was performed.

In addition to internal consistency, test–retest reliability was assessed to evaluate temporal stability. A statistically significant agreement was observed between the scale scores obtained at two different time points (ICC = 0.653), indicating moderate temporal stability.

Finally, several limitations related to reliability and generalizability should be acknowledged. The study sample consisted of undergraduate students from a single public university, with a predominance of female participants, which may limit the generalizability of the findings to broader demographic and cultural groups.

### 4.4 Comparison With Existing Measurement Tools

In recent years, several measurement tools have been developed to assess different dimensions of the relationship between climate change and health. However, these instruments differ substantially in terms of their conceptual focus, target populations, and intended outcomes. Within this expanding measurement landscape, the ClimHealth‐K scale contributes by addressing a distinct and relatively underexplored construct, namely individuals' perceived knowledge and awareness of the health effects of climate change, with a specific focus on university students.

One of the most comprehensive instruments in this field is the Climate Change Health Literacy Scale developed by Nayir et al. (2025), which is grounded in the health literacy framework and integrates knowledge, attitudes, and health‐related behaviors across four subdimensions, including health impact, monitoring, behavioral, and protection support dimensions. While this multidimensional structure enables a comprehensive assessment of climate‐related health literacy in the general adult population in Türkiye, the ClimHealth‐K differs in scope and intended construct. Rather than measuring functional health literacy or behavioral engagement, the ClimHealth‐K focuses specifically on self‐perceived knowledge and awareness regarding climate health interactions, capturing a more proximal cognitive construct that is theorized to precede attitude formation and behavior change (Nayir et al. 2025).

Similarly, the Climate Change Perceptions Scale for Health and Related Professionals and Students emphasizes perceptions, beliefs, and professional viewpoints related to climate change and health within health‐related disciplines (Sönmez et al. 2025). Although this scale provides valuable insights into professional orientations and perception‐based responses, it differs conceptually from the ClimHealth‐K, which is neither profession‐centered nor perception‐focused. Instead, the ClimHealth‐K was designed as a brief, unidimensional instrument to quantify perceived knowledge and awareness in a manner applicable to a broader university student population across disciplines.

Other existing instruments target even more specific outcome domains. For example, the Climate Change Health Protection Behaviors Scale developed for adolescents focuses explicitly on protective and adaptive behaviors, rather than on knowledge or awareness (Kurt et al. 2024). In a similar vein, climate change anxiety scales primarily assess emotional and psychological responses, such as worry, distress, and anxiety associated with climate change, rather than cognitive understanding of health‐related impacts (Clayton and Karazsia 2020; Innocenti et al. 2021).

Within this context, the ClimHealth‐K addresses a methodological gap by isolating perceived climate health knowledge and awareness as a standalone construct. This focus is particularly relevant for educational and public health research, as perceived knowledge and awareness constitute critical antecedents for both attitude development and subsequent health‐protective behaviors. Moreover, by targeting university students, the ClimHealth‐K provides a useful instrument for evaluating educational interventions and curriculum‐based initiatives aimed at strengthening climate health awareness among future professionals across disciplines.

Taken together, the ClimHealth‐K complements rather than duplicates existing measurement instruments by offering a concise, unidimensional assessment of perceived climate health knowledge applicable to educational settings.

### 4.5 Limitations and Future Directions

Several limitations should be acknowledged. The study sample consisted of undergraduate students from a single public university with a predominance of female participants, which may limit the generalizability of the findings to broader demographic and cultural groups. The disciplinary scope of the expert panel was also limited. In addition, although exploratory and confirmatory factor analyses supported a stable structure, future studies using independent samples would further strengthen the evidence base. Another important limitation of the present study is that convergent and discriminant validity were not examined. Although the internal structure of the scale was supported through exploratory and confirmatory factor analyses, these methods do not provide evidence regarding the relationships between ClimHealth‐K and other theoretically related or unrelated constructs. Therefore, future studies are recommended to evaluate convergent validity by examining its associations with conceptually similar measures, and discriminant validity by testing its relationships with theoretically distinct constructs.

Another important consideration is that the ClimHealth‐K measures perceived knowledge rather than objective knowledge. While perceived knowledge is theoretically meaningful and relevant for understanding motivation and engagement, it may not directly correspond to factual understanding. The lack of reverse‐worded items in the scale may be considered a limitation regarding potential acquiescence bias. However, recent psychometric literature suggests that reverse‐worded items can introduce method effects and reduce factorial clarity, particularly in awareness and knowledge‐oriented scales. For this reason, positively worded items were preferred in order to maximize interpretability and structural coherence. Future research should therefore examine associations between ClimHealth‐K scores and objective knowledge measures, as well as investigate predictive validity in relation to attitudes, intentions, and health‐protective behaviors.

Future studies are encouraged to conduct cross‐cultural validation, adapt the scale for different age groups and professional populations, develop shorter forms for rapid assessment when needed, and apply the scale in intervention studies to evaluate changes in climate and health awareness over time.

In conclusion, the ClimHealth‐K is a valid, reliable, and practically applicable instrument for assessing perceived knowledge and awareness of the health effects of climate change. By addressing a clearly defined cognitive construct and complementing existing measurement approaches, the scale offers a valuable resource for educational, public health, and policy‐oriented research aimed at strengthening climate and health awareness and resilience.

## 5 Conclusions

The findings of this study demonstrate that the ClimHealth‐K is a valid and reliable measurement tool for assessing university students' perceived knowledge and awareness of the health impacts of climate change. Psychometric analyses supported a clear unidimensional structure consisting of 11 items, with no reverse‐scored items. Total scores range from 11 to 55, with higher scores reflecting higher levels of perceived knowledge and awareness regarding the health effects of climate change.

By focusing specifically on perceived knowledge and awareness as a distinct cognitive construct, the ClimHealth‐K offers a concise and practical instrument that complements existing multidimensional tools in the climate and health literature. Its brevity, conceptual clarity, and unidimensional structure make it particularly suitable for educational and research settings where rapid, practical, and reliable assessment is required. The scale therefore represents a useful contribution to efforts aimed at understanding and strengthening climate and health awareness among future professionals across disciplines.

## 6 Relevance for Clinical Practice

The ClimHealth‐K has potential applicability across a range of educational, community health, and public health contexts. Educators and public health professionals may use the scale to assess perceived climate health knowledge and to identify priority areas for educational and awareness‐raising initiatives. In community and public health education settings, the scale may assist nurses, dietitians, and other health practitioners in identifying gaps in perceived climate‐related health knowledge and in planning targeted educational programs.

Furthermore, integrating the ClimHealth‐K into public health education programs and academic curricula may contribute to strengthening climate and health awareness and informing preventive educational strategies. Future research should explore the use of the scale in diverse populations, examine its predictive validity in relation to attitudes and health‐protective behaviors, and evaluate its sensitivity to change in intervention and longitudinal studies.

## Author Contributions

**Taha Gökmen Ülger:** conceptualization, investigation, writing – original draft, methodology, validation, formal analysis, writing – review and editing. **Elif Gençer Şendur:** conceptualization, methodology, validation, writing – review and editing, writing – original draft, formal analysis. **Esra Tunçer:** , writing – original draft, writing – review and editing, methodology, validation, formal analysis. **İsa Çelik:** writing – review and editing, writing – original draft, investigation, methodology. **Murat Bektaş:** supervision, conceptualization, methodology, writing – original draft, writing – review and editing.

## Funding

This research received no specific grant from any funding agency in the public, commercial, or not‐for‐profit sectors.

## Ethics Statement

At the outset, written approval was obtained from Bolu Abant Izzet Baysal University Human Research Ethics Committee in Social Sciences (Protocol No: 2024/103) to conduct the study. The study was carried out in accordance with the principles of the Declaration of Helsinki. No data were collected that could reveal the personal identities or private information of the participating students. Google Forms was configured not to collect any personal data or email addresses.

## Consent

Informed consent was obtained from all participants prior to data collection. Participation was voluntary, and no identifying personal data were collected.

## Conflicts of Interest

The authors declare no conflicts of interest.

## Data Availability

The data that support the findings of this study are available from the corresponding author upon reasonable request.
